# Taurine inhibits *Streptococcus uberis-*induced NADPH oxidase-dependent neutrophil extracellular traps *via* TAK1/MAPK signaling pathways

**DOI:** 10.3389/fimmu.2022.927215

**Published:** 2022-08-25

**Authors:** Ming Li, Yabing Gao, Zhenglei Wang, Binfeng Wu, Jinqiu Zhang, Yuanyuan Xu, Xiangan Han, Vanhnaseng Phouthapane, Jinfeng Miao

**Affiliations:** ^1^ Ministry of Education Joint International Research Laboratory of Animal Health and Food Safety, Key Laboratory of Animal Physiology & Biochemistry, College of Veterinary Medicine, Nanjing Agricultural University, Nanjing, China; ^2^ National Research Center for Veterinary Vaccine Engineering and Technology of China, Jiangsu Academy of Agricultural Sciences, Nanjing, China; ^3^ Shanghai Veterinary Research Institute, Chinese Academy of Agricultural Sciences, Shanghai, China; ^4^ Department of Livestock and Fisheries, Ministry of Agriculture and Forestry, Vientiane, Laos

**Keywords:** taurine, *Streptococcus uberis*, neutrophil extracellular traps, NADPH oxidase, TAK1/MAPK

## Abstract

Neutrophil extracellular traps (NETs) are produced by neutrophil activation and usually have both anti-infective and pro-damage effects. *Streptococcus uberis* (*S. uberis*), one of the common causative organisms of mastitis, can lead to the production of NETs. Taurine, a free amino acid abundant in the organism, has been shown to have immunomodulatory effects. In this study, we investigated the molecular mechanisms of *S. uberis*-induced NETs formation and the regulatory role of taurine. The results showed that NETs had a disruptive effect on mammary epithelial cells and barriers, but do not significantly inhibit the proliferation of *S. uberis*. *S. uberis* induced NADPH oxidase-dependent NETs. TLR2-mediated activation of the MAPK signaling pathway was involved in this process. Taurine could inhibit the activation of MAPK signaling pathway and NADPH oxidase by modulating the activity of TAK1, thereby inhibiting the production of ROS and NETs. The effects of taurine on NADPH oxidase and NETs in *S. uberis* infection were also demonstrated *in vivo*. These results suggest that taurine can protect mammary epithelial cells and barriers from damage by reducing *S. uberis*-induced NETs. These data provide new insights and strategies for the prevention and control of mastitis.

## Introduction

Neutrophil extracellular traps (NETs) are extracellular web-like structures released by neutrophils after specific stimulation and are made up of a combination of DNA, histones, granule proteins, antimicrobial peptides and so on. The process of their release is called NETosis (1). It has been a hot topic of research in neutrophil biology due to its close association with pathological phenomena such as infectious inflammation, tumor behavior and tissue damage. In recent years, research on the link between NETs and mastitis has been gaining attention. Swain et al. found NETs only was detected in the milk of cows with clinical mastitis, but not subclinical mastitis (2, 3). The milk can affect the phagocytosis of pathogenic bacteria by neutrophils, but has no inhibitory effect on the formation of NETs (4). In addition, it has been suggested that NETs and their histone components can cause damage to mammary epithelial cells (5). These studies suggest that NETs may be related to the severity of mastitis. Therefore, understanding the mechanism of neutrophil NETosis and regulating it appropriately could help in the prevention and control of mastitis.

Different stimuli can induce NETs by different molecular mechanisms (6). NADPH oxidase, a key enzyme in redox signaling, is a major generator of reactive oxygen species (ROS) *in vivo*. ROS generation by NADPH oxidases have been shown to be involved in most of the mechanisms underlying NET induction. MAPK is an important cellular signal that regulates cellular immune defense, including extracellular signal-regulated kinase (ERK), p38 family of kinases, and c-Jun N-terminal kinases (JNK) (7). The MAPK signaling pathway has been reported to be associated with the formation of NADPH oxidase-mediated NETs induced by *Escherichia coli* (*E. coli*), *Streptococcus lactis* and *Streptococcus suis* (8–10). These studies suggest that the extent to which p38, ERK and JNK affect the formation of NETs is pathogen-specific. *Streptococcus uberis* (*S. uberis*) is by far the most common causative agent of *Streptococcus* mastitis, with increasing prevalence worldwide (11, 12). Reinhardt et al. reported the observation of NETs formation in *S. uberis*-infected mammary glands (13). However, it is unclear whether MAPK signaling pathway and NADPH oxidase are involved in the mechanisms underlying *S. uberis*-induced NETs.

Nutritional modulation has been shown to be a viable strategy for mastitis prevention and control. Taurine, as the most abundant free amino acid in the body, has anti-inflammatory, antioxidant, ion homeostasis and metabolic regulating functions (14). Neutrophils are important effector cells in the mammary gland inflammatory response. Taurine, present in neutrophils at levels of up to 20-50 mM, has a regulatory effect on the immune function of neutrophils (15). For example, taurine supplementation *in vitro* has been shown to down-regulate pro-inflammatory-related gene expression in bovine PMN and to improve cellular antioxidant properties (16). Our group has shown that taurine can alleviate mammary inflammation and protect the integrity of the blood-milk barrier in mice infected with *S. uberis*, indicating the regulatory effect of taurine on the mammary inflammatory response (17). Based on this, understanding the regulatory effect of taurine on NETosis will contribute to furthering reveal the protective effect of taurine on the mammary gland in *S. uberis* infection.

In the present study, we aimed to explore the molecular mechanisms of NETosis in *S. uberis* infection and the role of taurine. The data clearly demonstrated that *S. uberis* induced NADPH oxidase-dependent NETs *via* TLR2/TAK1/MAPK signaling pathway. Taurine was able to limit NETs formation by inhibiting TAK1 activity. This helps to reduce the disruption of the mammary epithelial barrier by NETs. These data provide new scientific basis for the use of taurine in the prevention and control of mastitis.

## Materials and methods

### Bacterial culture

*S. uberis* 0140J (ATCC, Manassas, USA) were inoculated into Todd-Hewitt broth (THB) supplemented with 2% fetal bovine serum in an orbital shaker at 37°C for 3-4 h until grown at an OD_600_ of 0.5-0.6 (about 1×10^9^ CFU/mL).

### Animals and model of mammary infection

Specific pathogen-free (SPF) C57BL/6 mice(WT-B6) were purchased from Nanjing Qinglongshan Animal Farm (Nanjing, China) and bred under specific pathogen-free conditions in the Nanjing Agricultural University Laboratory Anima Center. All procedures involving animals were approved by the committee on the Use and Care of Animals of Nanjing Agricultural University (Nanjing, China).

24 pregnant mice aged 8-10 weeks were randomly divided into 4 groups. Two groups (Taurine, Taurine+*S. uberis*) were treated with taurine. Mice received 200 mg/kg of taurine daily, suspended in physiological saline by intragastric gavage from gestation day 14 until parturition. The other groups (Control, *S. uberis*) were given only the equal volume of physiological saline. At 48 h after parturition, mice in the *S. uberis* and taurine+*S. uberis* groups were infused with approximately 1×10^7^ CFU/mL *S. uberis* in 50 μL of sterile pyrogen-free saline into the L4 and R4 teats and 50 μL of saline was given to the control and taurine group. In detail, after administration of ether anesthesia, the L4 and R4 teats of mice were moistened with 75% ethanol, and a 33-gauge needle fitted to a 1 mL syringe was gently inserted into the mammary duct, then 50 μL of *S. uberis* or physiological saline was injected. At 24 h post infection, all mice in the 4 groups were euthanized, and the mammary gland and blood serum were collected and stored at -80°C until analyzed.

### EpH4-Ev cells culture

EpH4-Ev cells (ATCC, Manassas, USA) were grown in Dulbecco’s modified Eagle’s medium (DMEM, Gibco, NY, USA) with 10% fetal bovine serum (FBS, Gibco, NY, USA) in 6-well plates until the confluence reached 70–80%.

### Bone marrow neutrophil extraction and treatment

Neutrophils were isolated from 6-8 week old C57BL/6 mice(WT-B6) bone marrow as previously described (18). Tibias and femurs were collected from euthanized mice. Bone marrow were suspended in PBS buffer before overlaid on discontinuous percoll gradients (55%, 62%, and 81% in the order from top to bottom) (Solarbio, Beijing, China). After centrifugation at 1,000× g for 30 min, cells at the interface between 62% and 81% gradients were harvested and washed by 1640 medium. The cells were cultured in 1640 medium (Gibco, NY, USA) with 5% heat-inactivated FBS in a constant temperature cell incubator at 37°C and 5% CO_2_. Cells were used in subsequent experiments when they reached more than 85% Ly6G^+^ analyzed flow cytometry.

In the challenge experiments, the *S. uberis* grown at an OD_600_ of 0.5–0.6 was centrifuged for 10 min at 3,000× g and resuspended in an equal volume of PBS. Then this suspension was added to cells (MOI = 10). For the treatment experiments, neutrophils were pre-treated with taurine (Sigma, MO, USA), NAC (ROS scavenger, Beyotime, Nantong, China), DPI (NADPH oxidase inhibitor, Sellcek, TX, USA), mitoTEMPO (Mitochondria ROS scavenger, Cayman, MI, USA), Losmapimod (p38 inhibitor, TargetMOL, Shanghai, China), SCH772984 (ERK inhibitor, TargetMOL, Shanghai, China), SP600125 (JNK inhibitor, TargetMOL, Shanghai, China), NG25 (TAK1 inhibitor, Invitrogen, CA, USA) and TLR2 antibody (Affinity, OH, USA) 1 h before addition of *S. uberis*.

### Cell viability assay

The effect of taurine on neutrophils viability was determined by CCK-8 assay (Solarbio, Beijing, China). Briefly, the cells were seeded at 1×10^4^ cells/well in a 96-well plate and treated with different doses of taurine for 5 h. Then 10 μL of CCK-8 was added into each well and incubated for another 1 h. The optical density was measured at OD_450_.

### Fluorescent staining of NETs

The concentration of extracted neutrophils was adjusted to 5×10^6^/mL using 1640 medium containing 5% heat-inactivated FBS, and 500 μL of cell suspension was added to each well of a 24-well plate. After adding *S. uberis* (MOI = 10), mix thoroughly and then centrifuge using 1,000× g for 10 min to spin down cells. Neutrophils were incubated at 37°C, 5% CO2 for 4 h. After incubation, adding SYTOX GREEN (1 μM) staining solution to treat for 10 min and observe directly under a fluorescent microscope.

### NETs induction and quantification

The concentration of extracted neutrophils was adjusted to 5×10^6^/mL using 1640 medium containing 5% heat-inactivated FBS, and 500 μL of cell suspension was added to each well of a 24-well plate. After stimulation by *S. uberis* (MOI = 10), the medium was removed and then 0.5 mL of 1640 medium was added to the well plate. Wells were treated with 0.1 mg/mL DNase І (Solarbio, Beijing, China) and incubated at 37°C for 10 min to partially digest NETs. The cell suspension was transferred to a sterile centrifuge tube by centrifugation at 500× g for 10 min and the cell free dsDNA supernatant was collected.

NETs in the supernatant and blood serum were quantified by measuring the dsDNA content in the supernatant according to the Quant-iT™ PicoGreen ^®^ dsDNA kit instructions (Invitrogen, CA, USA).

### Collection of NETs

To perform induction and isolation of the NETs, 5×10^6^ neutrophils were stimulated for 4 h using 100 nM PMA (Solarbio, Beijing, China). The medium was removed and then 0.2 mL of DMEM was added to the well plate. Wells were treated with 0.1 mg/mL of DNase І and incubated at 37°C for 10 min to partially digest NETs. The cell suspension was transferred to a sterile centrifuge tube by centrifugation at 500× g for 10 min and the cell free dsDNA supernatant was collected. NETs in the supernatant were quantified by measuring the dsDNA content in the supernatant according to the Quant-iT™ PicoGreen ^®^ dsDNA kit instructions.

### LDH assays of EpH4-Ev cells

The lactate dehydrogenase (LDH) activity in EpH4-Ev cell supernatants were determined using commercial kits (Solarbio, Beijing, China) according to the manufacturer’s instructions.

### Measurement of mammary epithelial barrier permeability

EpH4-Ev cells were inoculated in the upper chamber of the Transwell (Millipore, MA, USA). The culture medium was changed 24 h after inoculation and every other day thereafter. The trans-epithelial cell resistance was measured using a Millicell resistivity meter. TEER value = (actual value - blank control assay)/bottom area of the chamber. The TEER increased significantly after about 5-7 d of cell culture, demonstrating the formation of a somatic mammary epithelial barrier model, and could be used for subsequent experimental studies when the TEER was > 1500 Ω/cm^2^. Then NETs or DNase І (3 mg/mL) were added in the upper chamber. FITC-dextran (FITC-D, Sigma, MO, USA) was selected as a marker for paracellular transport. After stimulated for 12 h, the monolayer was washed and 200 μL of 1 mg/mL FITC-D was added to the upper chamber. After incubation for 1 h at 37°C and 5% CO_2_, the sample from the lower chamber was collected. The fluorescence intensity of FITC-D in the samples (excitation wavelength 490 nm, emission wavelength 520 nm) was measured using a fluorescent enzyme marker.

### Determination of the antibacterial effect of NETs

About 1×10^3^ CFU/mL *S. uberis* 0140J was in sterilized DMEM medium containing 400 ng/mL NETs or DNase І (3 mg/mL) and incubated at 37°C for 4 h. Then the bacteria solution was diluted 10-fold with sterile saline in a gradient manner. The mixed dilution was evenly applied to a solid THB dish and incubated at 37°C for 24 h. Plates with CFU in the range of 30-300 were selected for counting and the number of viable colonies was calculated according to the dilution.

### ROS determination

Intracellular ROS of neutrophils was stained by DCFH-DA according to the instructions (Beyotime, Nantong, China). Briefly, 1×10^6^ neutrophils were infected with *S. uberis* 0140J at a MOI of 10 for 2 h, and then the cells were loaded with 10 μM DCFH-DA for 30 min at 37°C. Cells were washed 3 times with PBS. Next, they were collected at 1,000× g for 5 min and resuspended in PBS. The cell samples were immediately analyzed by flow cytometry using a FACSCanto instrument; 10 000 cells per sample were analyzed using CellQuest Pro acquisition software and FlowJo software.

### Western blot

Total proteins were isolated from mouse mammary gland and neutrophils with RIPA lysis buffe (Solarbio, Beijing, China) with added 1 mM phenylmethylsulfonyl fluoride (PMSF, Solarbio, Beijing, China). The supernatants were collected by centrifuging at 12,000 rpm for 10 min at 4°C. Protein concentration was measured by bicinchoninic acid assay (BCA) (Beyotime, Nantong, China). About 30 μg protein lysates were separated on polyacrylamide gel by electrophoresis and transferred onto polyvinylidene diﬂuoride (PVDF) membranes (Millipore, MA, USA). The membranes were blocked with 5% bovine serum albumin diluted in Tris buffered saline with Tween-20 (TBST) for 2 h at room temperature and hybridized overnight with primary antibody (1:1000) at 4°C. Before and after incubation with the HRP-linked anti-rabbit IgG (1:10000, CST, MA, USA) at room temperature for 2 h, the membranes were washed 3 times with TBST. The signals were detected by an ECL Western blot analysis system (Tanon, Shanghai, China). Analysis of bands was quantifed with ImageJ software (NIH, USA). The primary antibodies were listed as follows: β-actin (ABclonal, Wuhan, China), TAK1, p-TAK1 (CST, MA, USA), p38, p-p38, ERK, p-ERK, JNK, p-JNK, p47^phox^, p-p47^phox^ and TLR2 (Affinity, OH, USA).

### Statistical analysis

All experiments were repeated at least 3 times. Results were analyzed using the GraphPad Prism 8.0 software (La Jolla, CA, USA). Data were expressed as means ± standard error of the mean (SEM). Differences were evaluated by one-way analysis of variance (ANOVA) followed by Tukey’s tests and Student–Newman–Keuls test. Significant differences were *P* < 0.05.

## Results

### *S. uberis* induces NETs that cause damage to EpH4-Ev cells and mammary epithelial barrier damage

**Figure 1:**
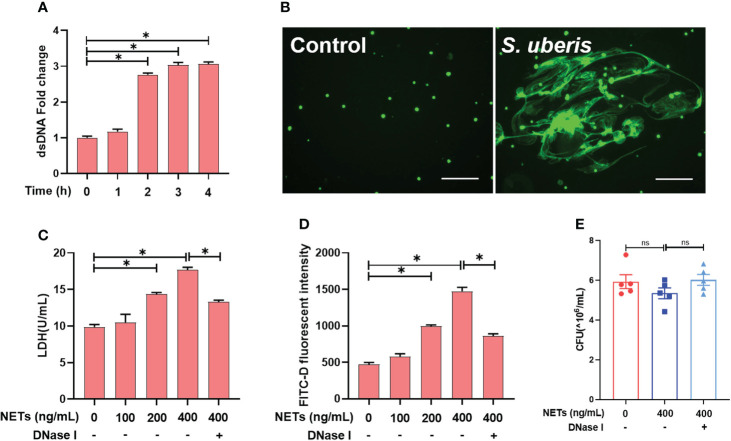
*S. uberis* induce NETs that cause damage to EpH4-Ev cells and mammary epithelial barrier damage. **(A, B)** Neutrophils were infected with *S. uberis* at MOI of 10. **(A)** The extracellular dsDNA content of neutrophils (n = 3). **(B)** SYTOX Green fluorescence staining of neutrophil extracellular dsDNA at 4 h after *S. uberis* stimulation (green) (n = 3). **(C, D)** EpH4-Ev cell and mammary epithelial barrier were treatment with NETs or DNase І (1.5 mg/mL) for 12 h. **(C)** LDH activity in EpH4-Ev cells supernatant (n = 3). **(D)** FITC-dextran (FITC-D) flux of mammary epithelial barrier (n = 3). **(E)** CFU of *S. uberis* (n = 5). Data are presented as mean ± SEM. *P > 0.05. ns, not significant (P > 0.05).

First, we found that *S. uberis* challenge of neutrophils for more than 2 h resulted in a significant increase in extracellular dsDNA (**Figure 1A**). By STYOX Green fluorescence staining, we observed the reticulated DNA backbone of NETs (**Figure 1B**). So the extracellular DNA levels were quantified to evaluate the NET formation. This suggests that *S. uberis* could induce NETs formation. Next, we treated mammary epithelial cells and mammary epithelial barriers with NETs for 12 h. Treatment with NETs at 200 ng/mL and 400 ng/mL resulted in a significant increase on LDH activity of EpH4-Ev cell supernatants as well as mammary epithelial barrier permeability compared to controls. In contrast, pretreatment with DNase І enzyme significantly reduced the damage to cells and barrier by NETs (**Figures 1C, D**). In addition, we also found that 400 ng/mL of NETs had no significant effect on the proliferation of *S. uberis* (**Figure 1E**). These results suggest that NETs cause EpH4-Ev cell damage and disrupt mammary epithelial barrier integrity, but may have no effect on *S. uberis* proliferation.

### NADPH oxidase-mediated ROS dominate the production of NETs in *S. uberis* infection

**Figure 2:**
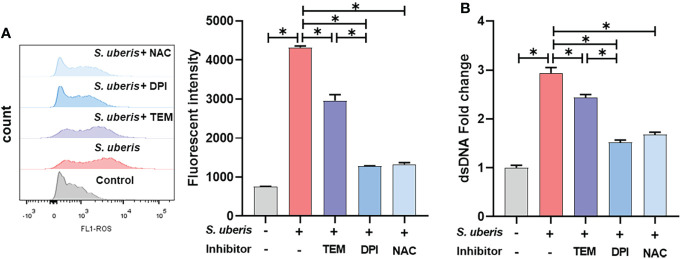
NADPH oxidase-mediated ROS dominate the production of NETs in *S. uberis* infection. The neutrophils were pretreated with NAC (5 mM), DPI (2 μM) and mitoTEMPO (TEM, 10 μM) for 1 h before infected with *S. uberis* at MOI of 10 for 2 h at 37°C. **(A)** The intracellular ROS level evaluated by Flow Cytometry. **(B)** The dsDNA content in supernatant. Data are presented as mean ± SEM (n = 3). *P < 0.05.

To investigate whether there is a link between *S. uberis*-induced NETs and ROS, NAC, DPI and mitoTEMPO were used to pretreat the cells. As shown, *S. uberis* significantly increased intracellular ROS levels compared to controls (**Figure 2A**). Pretreatment with NAC, mitoTEMPO or DPI significantly inhibited the *S. uberis*-induced rise in intracellular ROS and also significantly reduced the level of NETs in the supernatant. Moreover, DPI was significantly more effective than mitoTEMPO (**Figures 2A, B**). The results imply that NADPH oxidase-mediated ROS production in *S. uberis* infection is an important factor driving the formation of neutrophil NETs.

### MAPK signaling pathway is involved in *S. uberis*-induced NADPH oxidase activation and ROS production

**Figure 3:**
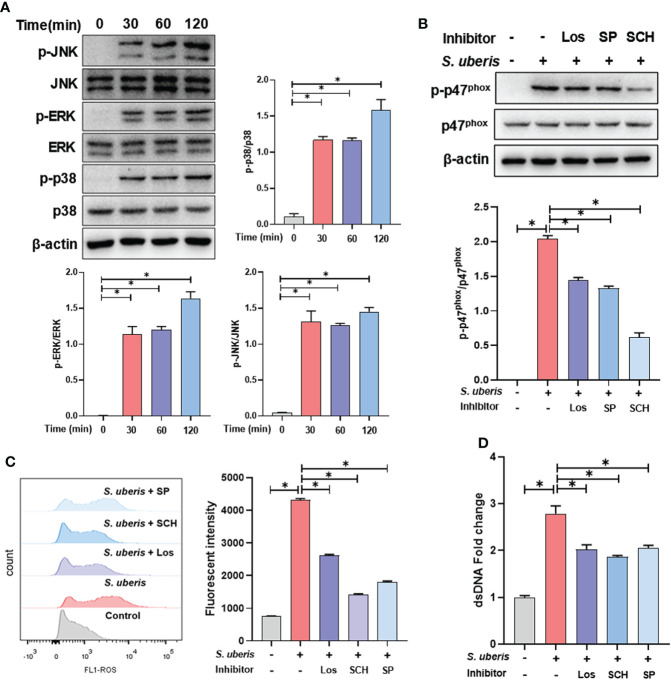
MAPK signaling pathway is involved in *S. uberis*-induced NADPH oxidase activation and ROS production. The neutrophils were pretreated with Losmapimod (Los, 20 μM), SCH772945 (SCH, 0.5 μM) and SP6001255 (SP, 10 μM) for 1 h before infected with *S. uberis* at MOI of 10. **(A)** Immunoblot and statistical analysis of neutrophil p38, ERK and JNK proteins and phosphorylated proteins. **(B)** Immunoblot and statistical analysis of neutrophil p47^phox^ proteins and phosphorylated proteins at 2 h. **(C)** The Intracellular ROS level evaluated by Flow Cytometry at 2 h. **(D)** The dsDNA content in supernatant at 4 h. Data are presented as mean ± SEM (n = 3). *P > 0.05.

To further clarify the upstream signals mediating NADPH oxidase activation, we examined the effect of *S. uberis* on MAPK signaling activation. We found that *S. uberis* caused a significant increase in the phosphorylation levels of p38, ERK and JNK in neutrophils (**Figure 3A**). Moreover, pretreatment with Losmapimod, SCH772945 or SP6001255 significantly reduced the levels of p47^phox^ phosphorylation and ROS (**Figures 3B, C**) and significantly downregulated the levels of NETs (**Figure 3D**). The results show that *S. uberis* induces NETs by activating NADPH oxidase production into ROS through the MAPK signaling pathway.

### TLR2 mediates the activation of MAPK signaling pathways in *S. uberis* infection

**Figure 4:**
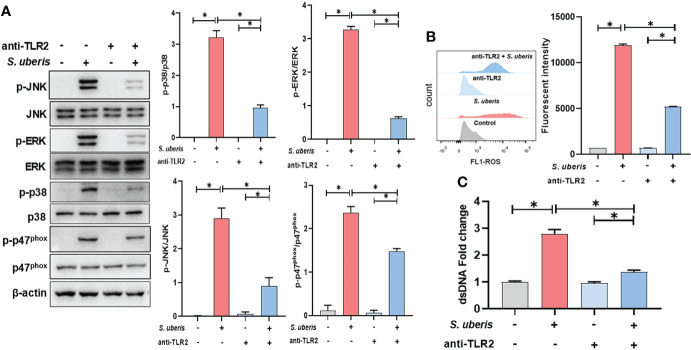
TLR2 mediates the activation of MAPK signaling pathways in *S. uberis* infection. The neutrophils were pretreated with TLR2-antibody (anti-TLR2, 100 ng/mL) for 1 h before infected with *S. uberis* at MOI of 10. **(A)** Immunoblot and statistical analysis of neutrophil p38, ERK, JNK and p47^phox^ proteins and phosphorylated proteins at 2 h. **(B)** The Intracellular ROS level evaluated by Flow Cytometry at 2 h. **(C)** The dsDNA content in supernatant at 4 h. Data are presented as mean ± SEM (n = 3). *P < 0.05.

To clarify whether TLR2 is involved in infection-induced MAPK signaling activation and NETosis, TLR2 antibodies were used to pretreat cells to block ligands from binding to TLR2. As shown in the figure, pretreatment with TLR2-containing antibodies significantly inhibited infection-induced increases in cellular p38, ERK, JNK and p47^phox^ protein phosphorylation levels (**Figure 4A**). At the same time, TLR2 antibody also caused a significant down-regulation of neutrophil intracellular ROS and supernatant NETs levels in infection (**Figures 4B, C**). This suggests that TLR2 mediates the activation of MAPK signaling and NADPH oxidase in neutrophils stimulated by *S. uberis*, contributing to NETs production.

### Taurine inhibits *S. uberis*-induced NETs production and MAPK signaling activation

**Figure 5:**
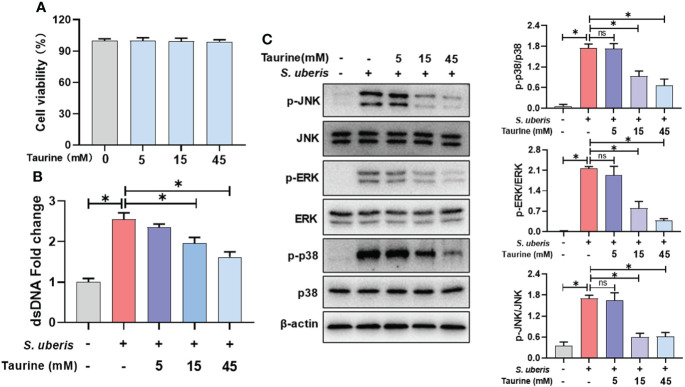
Taurine inhibits *S. uberis*-induced NETs production and MAPK signaling activation. The neutrophils were pretreated with taurine for 1 h before infected with *S. uberis* at MOI of 10. **(A)** The cell viabilities treated with taurine for 5 h detected by CCK-8 assay. **(B)** The dsDNA content in supernatant at 4 h. **(C)** Immunoblot and statistical analysis of p38, ERK and JNK proteins and phosphorylated proteins at 2 h. Data are presented as mean ± SEM (n = 3). *P > 0.05. ns, not significant (P > 0.05).

Next, we explored the role of taurine in regulating the release of neutrophil NETs in infection. Taurine treatment for 5 h had no significant effect on cell viability compared to the control group (**Figure 5A**). Compared to the *S. uberis* group, pretreatment with 15 mM and 45 mM taurine resulted in a significant reduction in NETs levels in supernatants (**Figure 5B**), and also significantly reduced cellular p38, ERK and JNK protein phosphorylation levels (**Figure 5C**). This suggests that MAPK signaling pathway is involved in the inhibitory effect of taurine on neutrophil NETosis in *S. uberis* infection.

### TAK1 is critical for the inhibition of MAPK signaling activation and NETs production by taurine in *S. uberis* infection

**Figure 6:**
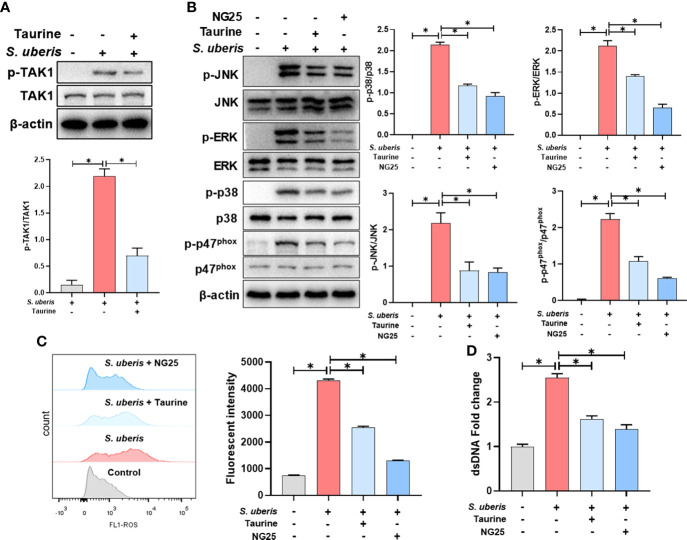
TAK1 is critical for the inhibition of MAPK signaling activation and NETs production by taurine in *S. uberis* infection. The neutrophils were pretreated with taurine (45 mM) or NG25 (40 nM) for 1 h before infected with *S. uberis* at MOI of 10. **(A)** Immunoblot and statistical analysis of TAK1 proteins and phosphorylated proteins. **(B)** Immunoblot and statistical analysis of p38, ERK, JNK and p47^phox^ proteins and phosphorylated proteins. **(C)** The Intracellular ROS level evaluated by Flow Cytometry. **(D)** The dsDNA content in supernatant. Data are presented as mean ± SEM (n = 3). *P < 0.05.

We further explored the regulatory mechanism of taurine on MAPK signaling pathway. Compared with the control group, TAK1 phosphorylation levels were significantly increased in the *S. uberis*-infected neutrophils. Taurine pretreatment significantly inhibited the phosphorylation level of neutrophil TAK1 in infection (**Figure 6A**). NG25, a TAK1 inhibitor, significantly inhibited the phosphorylation levels of p38, ERK, JNK and p47phox proteins, consistent with the effect of taurine (**Figure 6B**). Also, taurine and NG25 had a similar inhibitory effect on the production of neutrophil ROS and NETs (**Figures 6C, D**). These results suggest that taurine inhibits MAPK signaling pathway and NADPH oxidase activation by modulating TAK1 activity, thereby reducing the production of neutrophil ROS and NETs.

### Taurine inhibits NAPDH oxidase activity and NETs production in *S. uberis*-induced mastitis in mice

**Figure 7:**
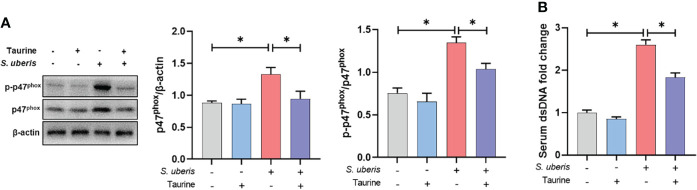
Taurine inhibits NAPDH oxidase activity and NETs production in *S. uberis-*induced mastitis in mice. **(A)** Immunoblot and statistical analysis of p47^phox^ proteins and phosphorylated proteins. **(B)** The dsDNA change in serum. Data are presented as mean ± SEM (n = 6). *P < 0.05.

Finally, we demonstrated the effect of taurine on neutrophil NETs production *in vivo* through a *S. uberis*-induced mice mastitis model. Taurine treatment significantly inhibited the increase in p47^phox^ protein expression and phosphorylation levels in mammary tissue resulting from *S. uberis* infection (**Figure 7A**). This is consistent with the results of the *in vitro* assay. At the same time, *S. uberis* caused a significant upward shift in the concentration of NETs in the serum of mice compared to the control group, while taurine significantly reduced the level of NETs (**Figure 7B**). This indicates that taurine has the same inhibitory effect on the production of NETs *in vivo*.

## Discussion

Mastitis is an important disease that threatens the development of dairy farming. *S. uberis* is a common causative agent of mastitis and is capable of inducing NETs formation. In this study, we found that *S. uberis* induced NADPH oxidase-dependent NETs *via* TLR2/MAPK signaling. NETs are a double-edged sword for the host’s immune defense system, which have been shown to cause damage to mammary epithelial cells. Here, the resultant data demonstrate taurine, a well-known nutrient, was able to inhibit NETosis by modulating the activity of TAK1 in *S. uberis* challenge. These data bring new insights into the prevention and control of mastitis.

Previous studies have found that most mammary pathogenic bacteria could induce NETosis (19). Such as *E. coli*, *Staphylococcus aureus* (*S. aureus*) and *Klebsiella pneumoniae* (19). Pisanu et al. detected NETs in the mammary gland alveoli and milk from *S. uberis*-infected sheep (20). In this study, we found that *in vitro S. uberis* stimulation of neutrophils induced NETs. It is well known that NETs usually have a dual role of being anti-infective and causing damage (1). The results of the present study showed that NETs at 200 and 400 ng/mL were able to cause damage to mammary epithelial cells, which is consistent with the findings of Wei et al (5). Moreover, we further found that NETs were able to disrupt the integrity of the mammary epithelial barrier, leading to increased permeability. However, the inhibitory effect of 400 ng/mL of NETs on *S. uberis* proliferation was not significant, suggesting that *S. uberis* may escape killing by NETs. It is not uncommon for pathogenic bacteria to escape killing by NETs. Many pathogenic bacteria have developed strategies to evade neutrophil immune defense mechanisms. For example, *S. aureus* can escape by secreting micrococcal nucleases to degrade NETs (21). Group A *Streptococci* have developed several strategies to evade NETs-dependent host defenses, including the expression of nucleases to degrade NETs (22, 23) and the inhibition of MPO release *via* M1T1 serotype *Streptococcal collagen*-like proteins (24). Indeed, it has been shown earlier that neutrophils have a limited role in host resistance to *S. uberis* infection and that the rise in neutrophil numbers induced by *S. uberis* infection failed to reduce CFU of bacteria in milk (25, 26). However, studies on the escape of *S. uberis* from killing by NETs have not been reported and the mechanism needs to be further investigated. In conclusion, the above results suggest that *S. uberis* is able to stimulate NETosis and may escape killing by NETs, implying that NETs may not play a role in the clearance of pathogenic bacteria in *S. uberis*-infected mastitis, but still cause damage to the mammary epithelial barrier. Therefore, limiting the formation of NETs is beneficial in alleviating the blood-milk barrier damage caused by *S. uberis* infection.

There are multiple ways in which neutrophils are affected by different stimuli to trigger NETosis, of which the typical triggering mechanisms can be categorized as NADPH oxidase-independent and NADPH-dependent pathways (6). The former is due to the transfer of large amounts of peptidylarginine deiminase 4 from the cytoplasm of neutrophils to the nucleus after binding to calcium ions, mediating histone periguanylation and subsequently promoting chromatin relaxation and release. The process is not dependent on the activation of NADPH oxidases. For example, *S. aureus*, *Candida albicans*, MIP-2, A23187 and ionomycin can induce NETosis through this mechanism (18, 27–29). The NADPH-dependent pathway is attributed to the activation of NADPH oxidase in neutrophils followed by the production of ROS, which then disassemble the nuclear membrane, allowing elastin and MPO to interact with the nucleus, thereby cleaving histones and promoting chromatin depolymerization. Eventually the neutrophil membrane is completely lost and the granular contents of the depolymerized DNA are released into the extracellular environment. Stimuli that trigger this mechanism include PMA, Oxidized LDL, *Pseudomonas aeruginosa* and so on (30–32). Here, DPI, mitoTEMPO and NAC all significantly reduced the *S. uberis*-induced increase in ROS levels and NETs release. Notably, NADPH oxidase inhibitors (DPI) were significantly more effective than mitochondrial ROS scavengers (mitoTEMPO), implyting that NADPH oxidase-mediated ROS play a dominant role in *S. uberis*-induced NETs. This suggests that *S. uberis* is capable of inducing NADPH oxidase-dependent NETs.

Our previous experiments showed that *S. uberis* induced neutrophil infiltration and MAPK signaling pathway activation in mammary tissue (17). Here, we also found that *S. uberis* stimulated JNK, p38 and ERK signaling activation in neutrophils. NADPH oxidase is a multi-component enzyme system that is active only after the assembly of four cytoplasmic proteins, p47^phox^, p67^phox^, p40^phox^ and Rac2, with the transmembrane proteins p22^phox^ and gp91^phox^. Phosphorylation of p47^phox^ at Ser379 is required for its activation (33). MAPK signaling pathway is one of the important pathways that induce NADPH oxidase activation and NETs formation (34). For example, JNK activation turns on LPS and *E. coli*-induced NADPH oxidase-dependent suicidal NETosis (10). NADPH oxidase-derived reactive oxygen species production activates the ERK1/2 pathway in *Streptococcus agalactiae*-induced NETs formation (8). *Streptococcus Suis* serotype 2 stimulates NETs formation *via* activation of both p38 and ERK1/2 (9). In this study, inhibition of p38, ERK and JNK all prevented *S. uberis*-induced phosphorylation levels of the p47^phox^ and reduced the production of ROS and NETs. In addition, TLR2 has an important role in the innate immune response of mammary tissue and mammary epithelial cells induced in *S. uberis* infection (7, 35). Here, the use of neutralizing antibodies to block TLR2 was able to reduce MAPK signaling and NADPH oxidase activation levels and decrease NETs formations. These results suggest that TLR2-mediated activation of the MAPK signaling pathway and NADPH oxidase leads to NETosis in *S. uberis* infection.

Because of the damaging effects of NETs, a growing number of studies have identified NETs as a therapeutic target for inflammatory diseases (36). Wang et al. showed that treatment with the peptidyl arginine deiminase inhibitor Cl-amidine could inhibit the release of NETs to reduce LPS-induced pathological damage in mouse mammary glands (37). Antioxidants are often used as NETosis modulators due to the close association of ROS with NETosis (33). Taurine is involved in the maintenance of neutrophil redox homeostasis (15). It has been reported taurine has prorotective effect on respiratory burst activity of polymorphonuclear leukocytes in endotoxemia (27). Abdelmegeid et al. showed that taurine supplementation down-regulated the expression of inflammation-related genes in bovine neutrophils (16). *In vitro* taurine exogenous taurine reduced the production of NETs stimulated by PMA and HOCl (38). Moreover, its derivative TauCl could also inhibit the production of NETs under PMA stimulation (39). We found 15 mM and 45 mM taurine pretreatment significantly inhibited *S. uberis*-induced NETs. At the same time, taurine also inhibited MAPK signaling pathway activation, consistent with the results of previous animal experiments (17). TAK1 is a member of the mitogen-activated protein kinase kinase family and is functionally located downstream of TLR2 (40). Further studies revealed that taurine was able to reduce the activation of TAK1. The inhibitory effects of TAK1 inhibitor (NG25) on MAPK signaling and NADPH oxidase activation were similar to those of taurine. These results suggest that taurine is able to inhibit MAPK/NADPH oxidase activation by modulating TAK1 activity in neutrophils, thereby limiting of NETs formation.

Our previous studies have demonstrated the ability of taurine to inhibit MAPK signaling activation in mice mammary tissue caused by *S. uberis* infection and to protect the blood-milk barrier (17). Here, taurine inhibited the expression of the p47^phox^ in *S. uberis*-infected mammary tissue, consistent with previous findings in a rat animal model (41). As a brief addition, we further found that taurine also had an inhibitory effect on the phosphorylation of the p47^phox^ which reflect NADPH oxidase activity and was able to significantly reduce the concentration of NETs in serum. It is important to note that these data do not prove that taurine has a direct effect on neutrophil NADPH activation and NETosis *in vivo*. Because a reduction in neutrophil trafficking to the gland, which has been shown by our previous studies (17), would also contribute to this result. However, it is sufficient to suggests that taurine may protect the blood-milk barrier from disruption by reducing NADPH oxidase activity and NETs formation.

In summary, *S. uberis* induced the formation of NETs by activating the neutrophil TLR2/TAK1/MAPK signaling pathway to drive NADPH oxidase production of ROS. Taurine was able to inhibit MAPK/NADPH oxidase activation by regulating TAK1 activity. Limiting effect of taurine on NETs contributes to reducing the disruption of the mammary epithelial barrier.

## Data availability statement

The original contributions presented in the study are included in the article/Supplementary Material. Further inquiries can be directed to the corresponding author.

## Ethics statement

All procedures involving animals were approved by the committee on the Use and Care of Animals of Nanjing Agricultural University (Nanjing, China).

## Author contributions

ML performed the whole experiments and wrote the manuscript. YG and ZW participated in the design of this study. BW and JZ provided assistance for data acquisition, data analysis, and statistical analysis. YX collected important background information. XH provided the support platform and funding. VP performed manuscript review. JM carried out the definition of intellectual content and provided the support platform and funding. All authors read and approved the final manuscript.

## Funding

This project was supported by grants from the Shanghai Agriculture Applied Technology Development Program, China (No. 2020-02-08-00-08-F01489); the National Natural Science Foundation of China (No. 32072867 and 31772701), the Special Fund for Independent Innovation of Agricultural Science and Technology in Jiangsu Province of China (No. cx (20) 3157), Key Scientific and Technological Project of XPCC (No. 2020AB025), the Key Project of Inter-governmental International Scientific and Technological Innovation Cooperation (No. 2018YFE0102200), Postgraduate Research & Practice Innovation Program of Jiangsu Province (KYCX22-0781, SJCX21-0240) and the Project Funded by the Priority Academic Program Development of Jiangsu Higher Education Institutions.

## Acknowledgments

The authors express their thanks to Dr. Howard Gelberg (Oregon State University) for manuscript editing.

## Conflict of interest

The authors declare that the research was conducted in the absence of any commercial or financial relationships that could be construed as a potential conflict of interest.

## Publisher’s note

All claims expressed in this article are solely those of the authors and do not necessarily represent those of their affiliated organizations, or those of the publisher, the editors and the reviewers. Any product that may be evaluated in this article, or claim that may be made by its manufacturer, is not guaranteed or endorsed by the publisher.
